# Dual Quaternion-Based Forward and Inverse Kinematics for Two-Dimensional Gait Analysis

**DOI:** 10.3390/jfmk10030298

**Published:** 2025-08-01

**Authors:** Rodolfo Vergara-Hernandez, Juan-Carlos Gonzalez-Islas, Omar-Arturo Dominguez-Ramirez, Esteban Rueda-Soriano, Ricardo Serrano-Chavez

**Affiliations:** Basic Sciences and Engineering Institute, Autonomous University of the State of Hidalgo, Pachuca 42184, Hidalgo, Mexico; ve138668@uaeh.edu.mx (R.V.-H.); omar@uaeh.edu.mx (O.-A.D.-R.); estebanrs@uaeh.edu.mx (E.R.-S.); se163352@uaeh.edu.mx (R.S.-C.)

**Keywords:** dual quaternions, forward kinematics, gait analysis, inverse kinematics, sagittal plane

## Abstract

**Background:** Gait kinematics address the analysis of joint angles and segment movements during walking. Although there is work in the literature to solve the problems of forward (FK) and inverse kinematics (IK), there are still problems related to the accuracy of the estimation of Cartesian and joint variables, singularities, and modeling complexity on gait analysis approaches. **Objective:** In this work, we propose a framework for two-dimensional gait analysis addressing the singularities in the estimation of the joint variables using quaternion-based kinematic modeling. **Methods:** To solve the forward and inverse kinematics problems we use the dual quaternions’ composition and Damped Least Square (DLS) Jacobian method, respectively. We assess the performance of the proposed methods with three gait patterns including normal, toe-walking, and heel-walking using the RMSE value in both Cartesian and joint spaces. **Results:** The main results demonstrate that the forward and inverse kinematics methods are capable of calculating the posture and the joint angles of the three-DoF kinematic chain representing a lower limb. **Conclusions:** This framework could be extended for modeling the full or partial human body as a kinematic chain with more degrees of freedom and multiple end-effectors. Finally, these methods are useful for both diagnostic disease and performance evaluation in clinical gait analysis environments.

## 1. Introduction

The study of human gait is an interdisciplinary field that integrates principles of biomechanics, neurophysiology, and orthopedics to understand movement patterns during bipedal locomotion [1]. The use of technologies such as force platforms and wearable or marker-based and marker-less systems for gait analysis allows the quantification of spatiotemporal, kinematic, and kinetic parameters, essential for the evaluation of neuromuscular pathologies, musculoskeletal injuries, and therapeutic interventions [2]. Recent advances in machine learning algorithms and wearable and inertial sensors have improved the accuracy and accessibility of these analyses, facilitating their application in clinical and sports environments [3].

Kinematics is the branch of mechanics that deals with the geometry of motion of rigid bodies without considering the masses of those bodies or the forces that cause movement. Forward and inverse kinematics are fundamental problems in clinical and research biomechanics [4]. The forward kinematics (FK) problem involves determining the position and orientation of the end-effector of an articulated chain given a collection of known joint angles and segment lengths, while the inverse kinematics (IK) problem seeks to calculate the joint angles using the known position and orientation of the end-effector and segment lengths [5]. Differential kinematics concerns the relationship between velocities. The forward differential kinematics problem consists in the determination of linear and angular velocities of the end-effector from known joint velocities. The inverse differential kinematic problem looks for the opposite relationship. Differential kinematics is often used to solve the IK problem through the derivation of the Jacobian matrix. Kinematic gait analysis involves the analysis of joint angles and segment movements during walking; previous studies suggest that examination of kinematic differences during the gait cycle can provide information related to the function of the lower limb to recommend postural control strategies [6]. Kinematic analysis and gait characterization have been used in many fields, including path planing for biped walking robots and humanoids [7,8], development of robotic exoskeletons for gait assistance [9], age-based performance evaluation [10], chronic stroke assessment [11], or cerebral palsy rehabilitation [12].

The solution to the FK problem has been extensively documented in the literature. A detailed description of the method for solving this problem using homogeneous transformation matrices is provided in [13]. Although rotation matrices in 3D space have nine elements, only three of them are independent, corresponding to the three degrees of freedom (DoF). This redundancy may lead to computational inefficiency [14]. Other ways of representing rotations are described in [15], such as the Euler angles representation, which uses three independent parameters, or the axis–angle representation, which uses four parameters to express the rotation of a given angle with respect to an axis in 3D space. Although these representations reduce parameter redundancy, they must be converted into rotation matrices to perform forward kinematics calculations.

Many robotics tasks, such as object manipulation, path tracking, or viewpoint control, rely on inverse kinematics (IK). However, for open kinematic chains, such as the human lower limb model used in this work, the IK problem is often difficult to solve analytically, and when closed forms solutions can be found, they are seldom unique [16]. On the other hand, numerical algorithms such as Newton–Raphson, Jacobian methods, and damped least squares converge to a single solution for IK [17]. Metaheuristics have recently attracted a lot of attention in the pursuit of answers to challenging optimization issues in subjects such as inverse kinematics [18]. Artificial intelligence has already been used in combination with dual quaternions to analyze and eliminate accumulated errors in the FK and IK of a robot arm. This combination showed high efficiency and accuracy and has the important advantage of being able to obtain the robot arm parameters without knowing the robot’s structure in advance [19].

Recently, quaternions have emerged as a fundamental tool in the kinematic analysis of robots and biomechanical systems with complex chains and structures due to their computational efficiency and absence of singularities, overcoming limitations of representations based on Euler angles [20]. Quaternions have also been used efficiently for the kinematics and dynamics of rigid bodies [21,22]. Using the dual-quaternion exponential and logarithm, an efficient derivation of dual-quaternion forms of forward, differential, and inverse kinematics that eliminates the singularity is possible [23]. This approach offers a potential way to use planar quaternions to control the 3-DoF of planar parallel mechanisms based on forward kinematics in real time [24]. The most related work presents a method to solve the inverse kinematic problem for articulated chains using an iterative dual-quaternion and exponential mapping strategy [25]. However, the authors point out some important limitations: when the joints are very close to their angular limits, the computed angles are pushed back and forth repeatedly, increasing the time to converge to a solution. Also, because the joint angles have not been weighted or coupled, some solutions generate unnatural postures. In addition, there are still problems to be solved, such as the compensation of modeling errors in biomechanical systems using quaternions, the real-time implementation in embedded platforms, or the generalization of quaternion-based methods for soft robots with nonlinear deformations.

In addition to the above-mentioned problems related to quaternion-based kinematic modeling, very few studies, including our previous works, have focused on the use of this approach to model human gait [26,27]. In [28] an approach for data based prediction of rigid body movements is presented. The method uses a combination of data-based learning with a physically motivated neural network architecture and dual quaternions. The obtained results support the applicability and potential of the approach in terms of improving prediction performance.

Therefore, this study proposes a framework for the solution of forward and inverse kinematics problems applied to gait analysis in the sagittal plane of a 3-DoF kinematic model of the lower limb. This is guided by our overarching research question: How to model the kinematics of a lower limb during the gait cycle using dual quaternions for gait analysis in the sagittal plane? The main contribution of this work is the proposed framework to model and evaluate the motion of a lower limb during the gait cycle using dual quaternions, which can be extended to a kinematic chain with more DoF and multiple end-effectors.

## 2. Materials and Methods

**Figure 1:**
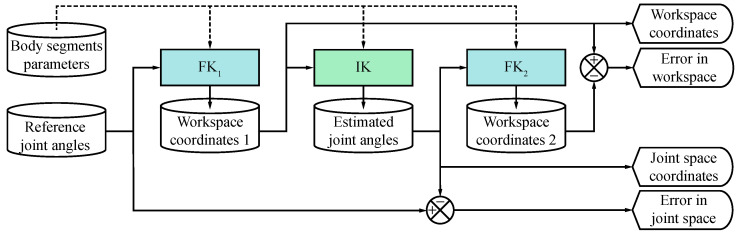
General framework for modeling a 3-DoF kinematic chain for two-dimensional gait analysis.

Figure 1 depicts the general framework for the 3-DoF human gait analysis used in this work. The framework consists of a sequence of processes to solve the forward and inverse kinematics problems for a kinematic chain representing a human lower limb. The first forward kinematic process (${\mathrm{FK}}_{1}$) has two inputs: the body segments parameters and the reference joint angles.

The input joint angles of the 3-DoF kinematic model consist of hip flexion–extension, knee flexion–extension, and ankle dorsiflexion–plantarflexion. With the objective to assess the accuracy of the IK method in different scenarios, we use three different gait cycles: normal gait, toe-walking, and heel-walking. The dataset of reference joint angles, publicly available and recorded using the SMART-E motion capture system (BTS, Milano, Italy), was acquired at 60 Hz during gait cycles performed by a group of subjects aged 22 to 72 years (mean 43.1±15.4), with a mean body mass of 68.5±15.8 kg and a mean height of 1.71±0.10 m [29].

The anatomical model used in this work corresponds to a subject with a height of 1.65 m. It is considered representative of the population of the dataset, since its height lies within one standard deviation from the mean height of the dataset population. The femur and tibia lengths, as well as lateral malleolus height and the distance from the lateral malleolus to the distal phalanx of the hallux (LM-to-DPH) were defined based on the adult human male anthropometric proportions [30] and measurements [31]. The body segments parameters are summarized in Table 1.

The ${\mathrm{FK}}_{1}$ solution is a set of coordinates that describe the positions of anatomical landmarks in the knee, ankle, and toe in the sagittal plane, as well as the orientations of the femur, tibia, and foot with respect to the global coordinate system (referred to as workspace coordinates 1). Next, using the workspace coordinates 1, an IK method is used to estimate a set of joint angles (designated as estimated joint angles) that best reproduce the corresponding positions and orientations of anatomical landmarks and body segments. The second forward kinematics process (${\mathrm{FK}}_{2}$) is then applied to the estimated joint angles to compute a second set of workspace coordinates (identified as workspace coordinates 2) for comparison purposes. To validate the results, RMS errors were calculated between the reference and estimated joint angles, as well as between workspace coordinates 1 and 2. Furthermore, all coordinates of the workspace and joint space were visualized and compared using 2D plots and a 3D model of the human right lower limb. A detailed description of each process, along with the fundamental theory, is provided in the following sections.

### 2.1. Quaternions Preliminaries

Quaternions were described by William Rowan Hamilton in 1843 [32]. Let H denote the set of quaternions. A quaternion q∈H is defined as

(1) $$q=q_{0}+q_{1}\vec{i}+q_{2}\vec{j}+q_{3}\vec{k}=q_{0}+\vec{q},$$

where $q_{0},q_{1},q_{2},q_{3}\in R$. The term $q_{0}$ is the real or scalar part and $\vec{q}=q_{1}\vec{i}+q_{2}\vec{j}+q_{3}\vec{k}\in R^{3}$ is the imaginary or vector part. The elements $\left\{\vec{i},\vec{j},\vec{k}\right\}$ are the unit vectors or imaginary units that satisfy $\vec{i^{2}}=\vec{j^{2}}=\vec{k^{2}}=\vec{i}\vec{j}\vec{k}=-1$ and form the orthonormal basis for $R^{3}$ [33]. Quaternions can also be represented as a column vector in $R^{4}$, that is

(2) $$q={\left[q_{0},\;q_{1},\;q_{2},\;q_{3}\right]}^{T}.$$

The following is a brief introduction to the essential definitions on quaternion and dual-quaternion algebra. For a comprehensive description of quaternion algebra refer to [34].

Given p,q∈H the following quaternion operations are defined:

- Addition. $p+q=\left(p_{0}+\vec{p}\right)+\left(q_{0}+\vec{q}\right)=\left(p_{0}+q_{0}\right)+\left(p_{1}+q_{1}\right)i+\left(p_{2}+q_{2}\right)j+\left(p_{3}+q_{3}\right)k$.
- Multiplication. $\left(p_{0}+\vec{p}\right)\left(q_{0}+\vec{q}\right)=p_{0}q_{0}-\vec{p}\;\cdot \;\vec{q}+\vec{p}\times \vec{q}+p_{0}\vec{q}+q_{0}\vec{p}$, where · and × denote the scalar and vector product in $R^{3}$, respectively [35]. Multiplication is associative and distributive but non-commutative.
- Multiplication identity. $1=1+\vec{0}$.
- Conjugate. $q^{*}=q_{0}-q_{1}\vec{i}-q_{2}\vec{j}-q_{3}\vec{k}=q_{0}-\vec{q}$.
- Magnitude. $\left|q\right|=\sqrt{qq^{*}}=\sqrt{q_{0}^{2}+q_{1}^{2}+q_{2}^{2}+q_{3}^{2}}\in R$.
- Inverse. $q^{-1}=\frac{q^{*}}{{\left|q\right|}^{2}}$, such that $qq^{-1}=q^{-1}q=1$.

There are two important subsets of H:

- $H_{1}\subset H$ is the set of unit quaternions defined as
  (3) $$H_{1}\equiv \left\{q\in H\;:|q|=1\right\}.$$
  This subset forms the 3-sphere $S^{3}\subset R^{4}$ and is used to represent 3D rotations. The inverse of a unit quaternion is its conjugate.
- $H_{p}\subset H$ is the set of pure quaternions defined as
  (4) $$H_{p}\equiv \left\{q\in H\;:q_{0}=0\right\}.$$
  Since the vector part of $q\in H_{p}$ is a column vector $\vec{q}\in R^{3}$, pure quaternions are commonly used to represent vectors and translations in 3D.

### 2.2. Rotations Using Unit Quaternions

**Figure 2:**
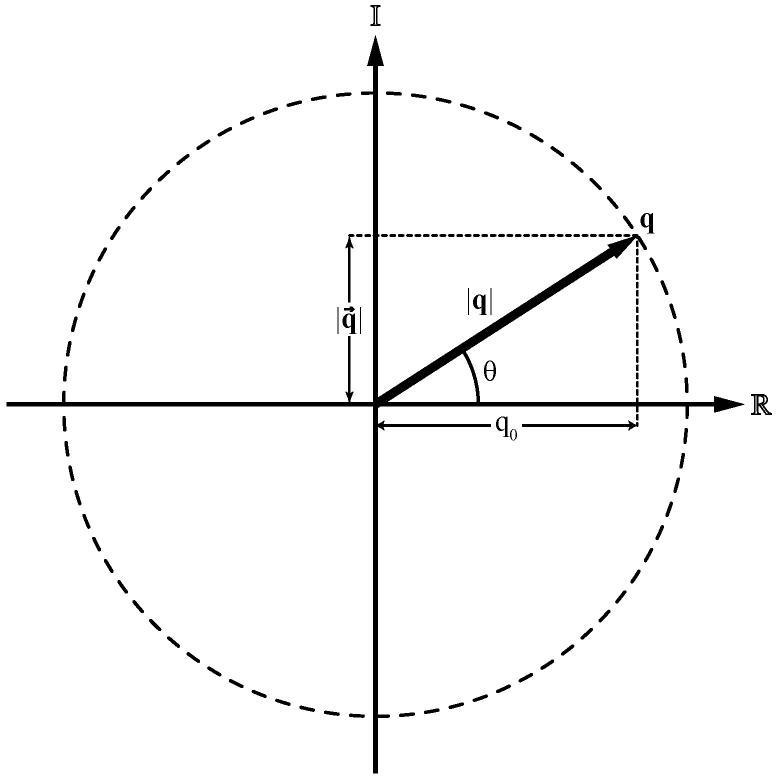
Representation of a unit quaternion as the radius of a unit circle in the complex plane. The coordinates are the scalar part $q_{0}$ and the norm of the vector part $|\vec{q}|$ in the R and I axes, respectively.

Unit quaternions can be treated as rotation operators in $R^{3}$. Figure 2 shows the projection of a unit quaternion in the complex plane, where it can be visualized as the radius of a unit circle. Let $q\in H_{1}$, and given that |q|=1, then

(5) $$q_{0}^{2}+{\left|\vec{q}\right|}^{2}=1.$$

Moreover, since for every angle θ, it is satisfied that $\cos^{2}\theta +\sin^{2}\theta =1$, there exists an angle θ∈(−π,π] such that $\cos\theta =q_{0}$ and $\sin\theta =|\vec{q}|$ [34]. Also, a unit vector $\vec{u}=\frac{\vec{q}}{|\vec{q}|}=\frac{\vec{q}}{\sin\theta }$ representing the rotation axis of q can be defined. Then, q can be written in terms of the angle θ and the unit vector $\vec{u}$ as

(6) $$q=q_{0}+\vec{q}=\cos\theta +\vec{u}\sin\theta .$$

The product of two unit quaternions that have the same rotation axis $\vec{u}$ is a unit quaternion that represents the sum of the two rotation angles about $\vec{u}$. Let $p=\cos\alpha +\vec{u}\sin\alpha \in H_{1}$ and $q=\cos\beta +\vec{u}\sin\beta \in H_{1}$, the quaternion product pq results in the following:

(7) $$pq=\cos\left(\alpha +\beta \right)+\vec{u}\sin\left(\alpha +\beta \right)\in H_{1}.$$

A unit quaternion $q\in H_{1}$ can be used to rotate a vector in $R^{3}$ by applying the transformation qvq*, where $v\in H_{p}$ is a pure quaternion that represents a 3D vector in the quaternion space. As an example, consider the unit quaternion $q=\cos\theta +\vec{k}\sin\theta$ representing a rotation of angle θ about the unit vector $\vec{k}$. Applying this rotation to the basis vector $\vec{i}$ gives the following:

(8) $$\begin{array}{cc} qiq^{*} & =\left(\cos\theta +\vec{k}\sin\theta \right)\left(0+\vec{i}\right)\left(\cos\theta -\vec{k}\sin\theta \right)\\ \; & =\vec{i}\cos2\theta +\vec{j}\sin2\theta \in H_{p}. \end{array}$$

Using the right-hand rule for rotation, the result is the vector $\vec{i}$ rotated counter-clockwise through an angle 2θ, about the vector $\vec{k}$ as an axis. Due to the fact that quaternion multiplication is non-commutative, the operation

(9) $$q^{*}iq=\vec{i}\cos2\theta -\vec{j}\sin2\theta \in H_{p},$$

results in the vector $\vec{i}$ rotated clockwise through an angle 2θ, about the vector $\vec{k}$ as an axis.

Extending Euler’s equation for the exponential map of a complex number to quaternions, a unit quaternion can be defined as the exponential map of an angle and a unit vector pair $\left(\theta ,\vec{u}\right)$. Let $\Phi =\Phi_{0}+\vec{\Phi }=0+\frac{1}{2}\theta \vec{u}\in H_{p}$, where, given the results of Equations (8) and (9), half the angle θ is used. The quaternion associated with this rotation is given by

(10) $$q=e^{\Phi_{0}+\vec{\Phi }}=e^{\Phi_{0}}e^{\vec{\Phi }}=\cos(|\vec{\Phi }\left|\right)+\frac{\sin(|\vec{\Phi }|)}{|\vec{\Phi }|}\vec{\Phi }$$

where $\vec{u}=\frac{\vec{\Phi }}{|\vec{\Phi }|}$ and $\frac{\theta }{2}=\left|\vec{\Phi }\right|$ [36]. However, when $|\vec{\Phi }|=0$, there is a singularity in $\frac{\sin(|\vec{\Phi }|)}{|\vec{\Phi }|}$ [23]. In order to avoid it, a Taylor series is used:

(11) $$\frac{\sin(|\vec{\Phi }|)}{|\vec{\Phi }|}\approx 1-\frac{|\vec{\Phi }{|}^{2}}{6}+\frac{|\vec{\Phi }{|}^{4}}{120}$$

On the other hand, the logarithm of a unit quaternion $q\in H_{1}$ that encodes a rotation of an angle $\frac{\theta }{2}$ about an axis $\vec{u}$ is given by

(12) $$\theta =2\;\cdot \;\arctan2(|\vec{q}|,q_{0}),$$

(13) $$\Phi =\frac{1}{2}\ln\left(q\right)=\frac{1}{2}\left(\ln\left(|q|\right)+\frac{\theta }{|\vec{q}|}\vec{q}\right)=0+\frac{1}{2}\theta \vec{u}\in H_{p}.$$

Note that, although the rotation angle θ could be recovered using $\theta =2\;\cdot \;\arccos q_{0}$, this formulation restricts the rotation to the interval 0≤θ<π. In contrast, using $\theta =2\;\cdot \;\arctan2(|\vec{q}|,q_{0})$ the possible rotation angles are in the range −π<θ≤π, ensuring proper treatment of positive and negative rotations. When $|\vec{q}|=0$, the expression $\frac{\theta }{|\vec{q}|}$ is not defined, and this is handled using a Taylor series as follows:

(14) $$\frac{\theta }{|\vec{q}|}=\frac{\frac{\theta }{\sin\theta }}{\left|q\right|}\approx \frac{1+\frac{\theta^{2}}{6}+\frac{7\theta^{4}}{360}}{\left|q\right|}$$

### 2.3. Dual Quaternions

Dual quaternions combine ordinary quaternions and dual numbers [23]. A dual quaternion is a number of the form

(15) $$\underline{q}=q_{p}+\varepsilon q_{d}\in H$$

where $q_{p}\in H$ is the primary part, $q_{d}\in H$ is the dual part, and ε is the dual operator satisfying $\varepsilon^{2}=0$. Given $\underline{p},\underline{q}\in H$, the following dual quaternion operations are defined:

- Multiplication. $\underline{p}\underline{q}=p_{p}q_{p}+\varepsilon p_{p}q_{d}+\varepsilon p_{d}q_{p}+\varepsilon^{2}p_{d}q_{d}=p_{p}q_{p}+\varepsilon \left(p_{p}q_{d}+p_{d}q_{p}\right)$.
- Conjugate. ${\underline{q}}^{*}=q_{p}^{*}+\varepsilon q_{d}^{*}$.
- Norm. $|\underline{q}|=\sqrt{\underline{q}{\underline{q}}^{*}}=\left|q_{p}\right|+\varepsilon \frac{q_{p}\;\cdot \;q_{d}}{|q_{p}|}$.
- Inverse. ${\underline{q}}^{-1}=\frac{{\underline{q}}^{*}}{|\underline{q}{|}^{2}}=q_{p}^{-1}-\varepsilon q_{p}^{-1}q_{d}q_{p}^{-1}$. Only when $q_{p}\neq 0$.

A dual quaternion is a unit if and only if $|q_{p}|=1$ and $q_{p}\;\cdot \;q_{d}=0$. The set of unit dual quaternions is denoted as $H_{1}\equiv \{\underline{q}\in H:|\underline{q}|=1\}$ [33]. Unit dual quaternions are always invertible because their inverse is their conjugate.

### 2.4. Rigid Transformations Using Dual Quaternions

Geometrically, elements of $H_{1}$ equipped with the multiplication operation represent rigid motions in Euclidian 3D space [33]. Let $r=e^{\frac{1}{2}\vec{u}\theta }\in H_{1}$ and $p=0+\vec{p}\in H_{p}$; the translation p followed by the rotation r is given by

(16) $$\underline{q}=\left(1+\varepsilon \frac{1}{2}p\right)r=r+\varepsilon \frac{1}{2}pr\in H_{1}.$$

Conversely, for any $\underline{q}=q_{p}+\varepsilon q_{d}\in H_{1}$ the rotation is given by $r=q_{p}\in H_{1}$, and the translation is given by

(17) $$p=2q_{d}{\left(q_{p}\right)}^{*}=prr^{*}\in H_{p}.$$

Remarkably, the composition of rigid transformations is given by a sequence of unit dual quaternion multiplications. Let ${\underline{q}}_{1},{\underline{q}}_{2}\in H_{1}$, thus

(18) $${\underline{q}}_{3}={\underline{q}}_{1}{\underline{q}}_{2}=\left(r_{1}+\varepsilon \frac{1}{2}p_{1}r_{1}\right)\left(r_{2}+\varepsilon \frac{1}{2}p_{2}r_{2}\right)=r_{1}r_{2}+\varepsilon \frac{1}{2}\left(r_{1}p_{2}r_{2}+p_{1}r_{1}r_{2}\right).$$

Note that the primary part of the result is the composition of the rotations as shown in Equation (7); that is

(19) $$q_{3_{p}}=r_{1}r_{2}\in H_{1},$$

whereas the composition of translations can be obtained from the dual part using Equation (17) as follows

$$p_{3}=2q_{3_{d}}q_{3_{p}}^{*}=\left(r_{1}p_{2}r_{2}+p_{1}r_{1}r_{2}\right)r_{2}^{*}r_{1}^{*}=p_{1}+r_{1}p_{2}r_{1}^{*}\in H_{p}.$$

### 2.5. Three-DoF Kinematic Model of the Human Lower Limb

**Figure 3:**
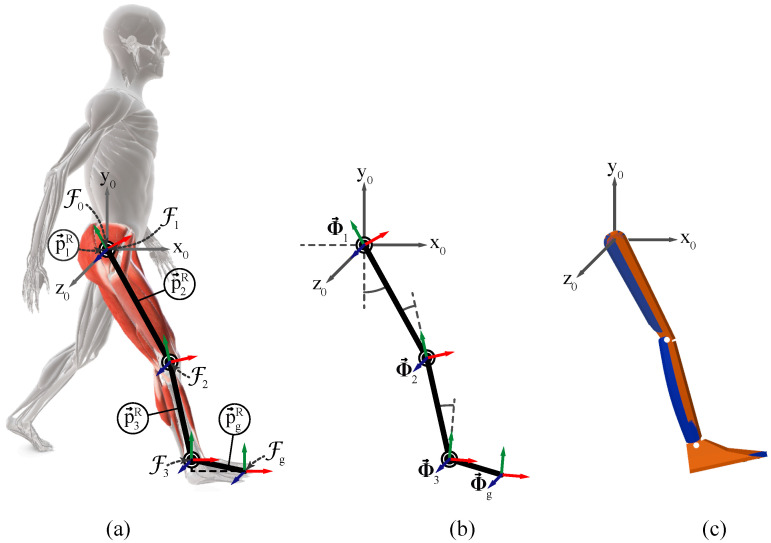
Right lower limb modeled as a 3-Degrees of Freedom (DoF) kinematic chain. (**a**) Joint local coordinate frames and body segment distances. (**b**) Axis–angle pair of each joint. (**c**) 3D model based on the body segment parameters. The *x*, *y*, and *z* axes of the local coordinate frames are indicated by the red, green, and blue arrows, respectively.

The biomechanical functions of the human body can be represented using a series of rigid bodies connected by joints. Figure 3 shows the collection of 3 rigid bodies connected by 3 revolute joints used to analyze the motion of the right lower limb in the sagittal plane. A revolute joint allows rotation around a single axis; therefore, each joint has one degree of freedom (DoF), while the resulting kinematic chain has three DoF.

The global coordinate system is represented by the fixed frame $F_{0}$ with coordinate axes $x_{0}$, $y_{0}$ and $z_{0}$ pointing in the anterior, cranial, and lateral (to the right) directions, respectively. The origin of $F_{0}$ is the origin of the kinematic chain and is also referred to as the frame of joint 0. The hip, knee, and ankle joints correspond to indices i={1,2,3}, respectively. These joints are associated with the femur, tibia, and foot segments, in that same order. Each joint is assigned a local coordinate frame, denoted by $F_{i}$, which defines the position of the anatomical landmarks at the joints and the orientation of the corresponding body segments. In anatomical posture all frames $F_{i}$ are aligned with frame $F_{0}$, this is the initial posture of the kinematic chain. Global coordinates, expressed with respect to $F_{0}$, are denoted by the superscript *W*, whereas relative coordinates, expressed with respect to the local coordinate frame of the preceding joint, are denoted by the superscript *R*.

Figure 3a illustrates that the distance from the origin of the global coordinate frame $F_{0}$ to the hip joint frame $F_{1}$ is expressed by ${\vec{p}}_{1}^{R}=\vec{0}$, since they are at the same point in space. The lengths of the femur and tibia are indicated by the *y* component of ${\vec{p}}_{i}^{R}\in R^{3}$ for i={2,3}, respectively, and correspond to the values listed in Table 1.

The symbol ${\vec{\Phi }}_{i}=\frac{1}{2}\theta_{i}{\vec{u}}_{i}$ in Figure 3b denotes the axis–angle pair at each joint (also known as the joint configuration), where $\theta_{1}$ is the hip flexion–extension angle, $\theta_{2}$ is the knee flexion–extension angle, and $\theta_{3}$ is the ankle dorsiflexion–plantarflexion angle. The rotation axis of each joint is given by ${\vec{u}}_{i}=\left[0,0,1\right]$. For the 3-DoF kinematic chain representing the lower limb, the chain configuration is given by

(20) $$\vec{\Phi }=\left\{{\vec{\Phi }}_{1},{\vec{\Phi }}_{2},{\vec{\Phi }}_{3}\right\}.$$

The anatomical landmark at the toe is considered the end-effector of the kinematic chain and is assigned the local coordinate frame $F_{g}$. The distance from $F_{3}$ to $F_{g}$ is represented by the *x* and *y* components of ${\vec{p}}_{g}^{R}\in R^{3}$ and take the values given in Table 1. The relative orientation between $F_{g}$ and $F_{3}$ is indicated by the rotation vector ${\vec{\Phi }}_{g}=\vec{0}$, since the orientation of the end-effector follows that of the ankle joint (joint 3).

### 2.6. Forward Kinematics

The posture (position and orientation) of any coordinate frame F in the global coordinate system is defined as

(21) $$\vec{\xi }=\left[{\vec{p}}^{W},{\vec{o}}^{W}\right]\in R^{6},$$

where ${\vec{p}}^{W}\in R^{3}$ and ${\vec{o}}^{W}\in R^{3}$ are the position and orientation vectors in $F_{0}$, respectively. Specifically, the positions are given by the 3D vectors ${\vec{p}}_{g}^{W}=\left[p_{g,x}^{W},p_{g,y}^{W},p_{g,z}^{W}\right]$ for the end-effector and ${\vec{p}}_{i}^{W}=\left[p_{i,x}^{W},p_{i,y}^{W},p_{i,z}^{W}\right]$ for the anatomical landmarks at the joints. The orientations are given by the 3D vector ${\vec{o}}_{g}^{W}=\left[o_{g,x}^{W},o_{g,y}^{W},o_{g,z}^{W}\right]$ for the foot and ${\vec{o}}_{i}^{W}=\left[o_{i,x}^{W},o_{i,y}^{W},o_{i,z}^{W}\right]$ for the femur (i=1) and the tibia (i=2). The forward kinematics problem is described formally by

(22) $$\vec{\xi }=f\left(\vec{\Phi }\right).$$

where *f* is a non-linear function that uniquely determines the posture of a given anatomical landmark F, based on the chain configuration vector $\vec{\Phi }$ up to that point.

Let $r_{i}^{R}\left({\vec{\Phi }}_{i}\right)=e^{{\vec{\Phi }}_{i}}\in H_{1}$ and $p_{i}^{R}=0+{\vec{p}}_{i}^{R}\in H_{p}$ be rotation and translation quaternions, respectively. The unit dual quaternion that represents the position and orientation of the local coordinate frame $F_{i}$ with respect to the local coordinate frame $F_{i-1}$ is given by

(23) $${\underline{q}}_{i}^{R}\left({\vec{\Phi }}_{i}\right)=\left(1+\varepsilon \frac{1}{2}p_{i}^{R}\right)r_{i}^{R}\left({\vec{\Phi }}_{i}\right)\in H_{1}.$$

The unit dual quaternion that represents the posture of the end-effector coordinate frame $F_{g}$ relative to the global coordinate frame $F_{0}$ can be found by

(24) $${\underline{q}}_{g}^{W}\left({\vec{\Phi }}_{i}\right)={\underline{q}}_{3}^{W}\left(\vec{\Phi }\right){\underline{q}}_{g}^{R}\left({\vec{\Phi }}_{g}\right)={\underline{q}}_{0}^{W}{\underline{q}}_{1}^{R}\left({\vec{\Phi }}_{1}\right){\underline{q}}_{2}^{R}\left({\vec{\Phi }}_{2}\right){\underline{q}}_{3}^{R}\left({\vec{\Phi }}_{3}\right){\underline{q}}_{g}^{R}\left({\vec{\Phi }}_{g}\right),$$

where

- ${\underline{q}}_{0}^{W}=1+0\in H_{1}$ indicates that joint 0 is fixed in the origin of the global coordinate frame.
- ${\underline{q}}_{3}^{W}\left(\vec{\Phi }\right)\in H_{1}$ describes the posture of joint 3 with respect to the global coordinate frame.
- ${\underline{q}}_{g}^{R}\left({\vec{\Phi }}_{g}\right)=\left(1+\varepsilon \frac{1}{2}p_{g}^{R}\right)r\left({\vec{\Phi }}_{g}\right)\in H_{1}$ denotes the posture of the end-effector with respect to the local coordinate frame of joint 3.

From Equation (18) it is known that ${\underline{q}}_{g}^{W}\left(\vec{\Phi }\right)=q_{g_{p}}^{W}+\varepsilon q_{g_{d}}^{W}=r_{g}^{W}+\varepsilon \frac{1}{2}p_{g}^{W}r_{g}^{W}\in H_{1}$. Using Equations (12), (13), and (17), the end-effector position ${\vec{p}}_{g}^{W}$ and orientation ${\vec{o}}_{g}^{W}$ can be extracted; therefore, the solution to the forward kinematics problem at any timestep of the gait cycle is given by

(25) $${\vec{\xi }}_{g}=\left[{\vec{p}}_{g}^{W},{\vec{o}}_{g}^{W}\right]=\left[2q_{g_{d}}^{W}{\left(q_{g_{p}}^{W}\right)}^{*},2\ln\left(q_{g_{p}}^{W}\right)\right].$$

Similarly, the forward kinematics solution for each coordinate frame $F_{i}$ is computed using Equation (24) by composing the transformations up to the *i*-th quaternion and is given by

(26) $${\vec{\xi }}_{i}=\left[{\vec{p}}_{i}^{W},{\vec{o}}_{i}^{W}\right]=\left[2q_{i_{d}}^{W}{\left(q_{i_{p}}^{W}\right)}^{*},2\ln\left(q_{i_{p}}^{W}\right)\right].$$

Algorithm 1 summarizes the method described for the calculation of the forward kinematics solutions [37].

**Algorithm 1** Forward kinematics**Require:** Body segments parameters ${\vec{p}}_{i}^{R}$, reference joint configurations ${\vec{\Phi }}_{i}$, and end-effector dual quaternion ${\underline{q}}_{g}^{R}\left({\vec{\Phi }}_{g}\right)$ n←numberofjoints **for**
i=1, i=n
**do**   $r_{i}^{R}\leftarrow e^{{\vec{\Phi }}_{i}}$   ${\underline{q}}_{i}^{R}\left({\vec{\Phi }}_{i}\right)\leftarrow \left(1+\varepsilon \frac{1}{2}p_{i}^{R}\right)r_{i}^{R}\left({\vec{\Phi }}_{i}\right)$   ${\underline{q}}_{i}^{W}\leftarrow {\underline{q}}_{i-1}^{W}{\underline{q}}_{i}^{R}$   ${\vec{p}}_{i}^{W}\leftarrow 2q_{i_{d}}^{W}{\left(q_{i_{p}}^{W}\right)}^{*}$   ${\vec{o}}_{i}^{W}\leftarrow 2\ln\left(q_{i_{p}}^{W}\right)$   ${\vec{\xi }}_{i}\leftarrow \left[{\vec{p}}_{i}^{W},{\vec{o}}_{i}^{W}\right]$ **end for** ${\underline{q}}_{g}^{W}\leftarrow {\underline{q}}_{n}^{W}{\underline{q}}_{g}^{R}$ ${\vec{p}}_{g}^{W}\leftarrow 2q_{g_{d}}^{W}{\left(q_{g_{p}}^{W}\right)}^{*}$ ${\vec{o}}_{g}^{W}\leftarrow 2\ln\left(q_{g_{p}}^{W}\right)$ **return**
${\vec{\xi }}_{g}\leftarrow \left[{\vec{p}}_{g}^{W},{\vec{o}}_{g}^{W}\right]$

### 2.7. Inverse Kinematics

Let *f* be the function that determines the end-effector posture vector ${\vec{\xi }}_{g}$ given the chain configuration vector $\vec{\Phi }$; the inverse kinematics problem can be defined as

(27) $$\vec{\Phi }\in f^{-1}\left({\vec{\xi }}_{g}\right),$$

which implies that there may not always exist a solution, and when it does, there may not be a unique solution [38]. Nevertheless, it is desirable to find a solution that results in the most natural posture and the most stable behavior. For the case of human lower limbs, this means finding a solution with knee-forward configuration and smooth trajectories. In some cases, it is possible to compute the solution $\vec{\Phi }$ analytically, by trying all possible joint configurations that bring the end-effector closer to the desired posture. For cases where it is not possible to compute an analytical solution, Jacobian-based methods iteratively approximate an effective inverse kinematics solution [25]. The basic equation for forward dynamics that describes the velocities of the end-effector is given by ${\dot{\vec{\xi }}}_{g}=J\dot{\vec{\Phi }}$, where *J* is the Jacobian matrix. This can be approximated as a small change in the end-effector posture caused by a small change in joint angles, given by

(28) $$\Delta {\vec{\xi }}_{g}\approx \frac{\partial {\vec{\xi }}_{g}}{\partial \vec{\Phi }}\Delta \vec{\Phi }\approx J\left({\vec{\xi }}_{g},\vec{\Phi }\right)\Delta \vec{\Phi }.$$

Therefore, the relationship expressed in Equation (27) can be approximated as

(29) $$\Delta \vec{\Phi }\approx J{\left({\vec{\xi }}_{g},\vec{\Phi }\right)}^{-1}\Delta {\vec{\xi }}_{g}.$$

In some cases, the Equation (29) cannot be solved uniquely. Indeed, the Jacobian may not be square or invertible, and even if is invertible, Equation (29) may work poorly if *J* is near singularity. A recurring problem in tracking target postures is that when the target postures are too distant, the kinematic chain stretches out to try to reach the target position. Once the chain is extended in this way, it is usually near a singularity, that is, the Jacobian is very sensitive to small changes in joint angles, and the chain may shake or jitter, attempting unsuccessfully to reach the distant target [38].

The Damped Least Squares (DLS) Jacobian method addresses many of the singularities problems of other Jacobian methods. DLS works by finding the value of $\Delta \vec{\Phi }$ that minimizes the value of

(30) $$∥J\Delta \vec{\Phi }-{\vec{\xi }}_{g}{∥}^{2}+\gamma^{2}∥\Delta \vec{\Phi }∥,$$

where γ is a positive, nonzero damping constant. The solution to the inverse kinematics problem using the DLS method is formally defined as

(31) $$\Delta \vec{\Phi }=J^{T}{\left(JJ^{T}+\gamma^{2}I\right)}^{-1}\Delta {\vec{\xi }}_{g}.$$

The value of the damping constant γ∈(0,1) depends on the parameters of the kinematic chain and target postures, which must be set to make the DLS numerically stable. Let $\Delta {\vec{\xi }}_{g}={\vec{\zeta }}_{g}-{\vec{\xi }}_{g}$ be the *error* between the current posture ${\vec{\xi }}_{g}$ and the target posture ${\vec{\zeta }}_{g}$ of the end-effector. By definition in Equation (29), the approximation $\Delta \vec{\Phi }$ is valid only for small changes $\Delta {\vec{\xi }}_{g}$. Therefore, it is convenient to approximate the end-effector posture ${\vec{\xi }}_{g}$ to the target posture ${\vec{\zeta }}_{g}$ only by a step size constant α∈(0,1). Then, the incremental posture update is defined as $\Delta {\vec{\xi }}_{g}=\alpha \left({\vec{\zeta }}_{g}-{\vec{\xi }}_{g}\right)$. Thus, the new configuration is as follows:

(32) $${\vec{\Phi }}^{\mathrm{new}}=\vec{\Phi }+\Delta \vec{\Phi },$$

and leads to a new posture of the end-effector ${\vec{\xi }}_{g}^{\mathrm{new}}=f\left({\vec{\Phi }}^{\mathrm{new}}\right)$. For ${\vec{\xi }}_{g}\in R^{6}$ and $\vec{\Phi }\in R^{9}$, the Jacobian matrix is given by

(33) $$J\left({\vec{\xi }}_{g},\vec{\Phi }\right)=\left[\begin{array}{c} J_{p}\left(\vec{\Phi }\right)\\ J_{o}\left(\vec{\Phi }\right) \end{array}\right]=\left[\begin{array}{ccc} \frac{\partial \xi_{g,1}}{\partial {\vec{\Phi }}_{1}} & \frac{\partial \xi_{g,1}}{\partial {\vec{\Phi }}_{2}} & \frac{\partial \xi_{g,1}}{\partial {\vec{\Phi }}_{3}}\\ \vdots & \vdots & \vdots \\ \frac{\partial \xi_{g,6}}{\partial {\vec{\Phi }}_{1}} & \frac{\partial \xi_{g,6}}{\partial {\vec{\Phi }}_{2}} & \frac{\partial \xi_{g,6}}{\partial {\vec{\Phi }}_{3}} \end{array}\right]\in R^{6\times 9},$$

where $\frac{\partial \xi_{g,k}}{\partial {\vec{\Phi }}_{i}}=\left(\frac{\partial \xi_{g,k}}{\partial \Phi_{i,1}},\frac{\partial \xi_{g,k}}{\partial \Phi_{i,2}},\frac{\partial \xi_{g,k}}{\partial \Phi_{i,3}}\right)$, for i={1,2,3}, k={1,2,…,6}, are the partial derivatives of the components of the posture vector of the end-effector with respect to the logarithm of $r_{i}^{R}\left({\vec{\Phi }}_{i}\right)$. In fact, the columns of the Jacobian matrix are given by

(34) $$\frac{\partial {\vec{\xi }}_{g}\left(\vec{\Phi }\right)}{\partial \vec{\Phi }}=\left[\frac{\partial {\vec{p}}_{g}^{W}\left(\vec{\Phi }\right)}{\partial \vec{\Phi }},\frac{\partial {\vec{o}}_{g}^{W}\left(\vec{\Phi }\right)}{\partial \vec{\Phi }}\right].$$

The calculation of the partial derivatives of ${\vec{p}}_{g}^{W}$ and ${\vec{o}}_{g}^{W}$ and of the Jacobian matrix is discussed extensively in [27]. At each timestep of the gait cycle, the inverse kinematics method iterates until the norm of the posture error $|{\vec{\zeta }}_{g}-{\vec{\xi }}_{g}|$ falls below a selected threshold ϵ, or when the number of iterations exceeds the maximum limit *N*.

### 2.8. IK Algorithm Parametrization

To prevent the IK solution from taking values outside the range of motion of the lower limb joints, it is necessary to set limits to the joint angles. Then, any rotation of the joints $\theta_{i}$ is clamped as follows:

(35) $$\theta_{i,\mathrm{clamp}}=\left\{\begin{array}{cc} \theta_{i,\max}, & \mathrm{if}\;\theta_{i}>\theta_{i,\max}\\ \theta_{i}, & \mathrm{otherwise}\\ \theta_{i,\min}, & \mathrm{if}\;\theta_{i}<\theta_{i,\min}, \end{array}\right.$$

where the angle $\theta_{i}$ is obtained from Equation (32) using $\theta_{i}=2\left|{\vec{\Phi }}_{i}^{\mathrm{new}}\right|$. The quaternion logarithm with the clamped angle is given by

(36) $${\vec{\Phi }}_{i,\mathrm{clamp}}^{\mathrm{new}}=\frac{1}{2}\vec{u}\theta_{i,\mathrm{clamp}}$$

Table 2 summarizes the angular limits of the joints used in this work [29].

Setting the angles of the the initial chain configuration ${\vec{\Phi }}^{0}$ with the knee in slight flexion ($-5^{\circ }$) forces a knee-forward solution. While setting the angles of the hip and the ankle joints close to the angle values in the first step of a given gait cycle improves the convergence time of the solution at the beginning of the estimation process. This is particularly visible in the heel-walking initial chain configuration values. The values shown in Table 3 were set heuristically.

The values of the algorithm parameters α and γ were computed by means of a particle swarm optimization algorithm [39,40]. Table 4, shows the numerical values of α and γ for each gait pattern used in this work.

Additionally, to determine the values of ϵ and *N*, the following was considered: The frequency sampling of the data provided by the authors is 60 Hz [29], which is equivalent to a period of $1.67\times 10^{-2}$ s. The resolution of the data was estimated as the minimum difference between all samples, for which the data were first sorted, and repeated values were removed. Then, the difference between contiguous values was calculated. Finally, the minimum difference was computed, the value of which was found to be $1.131\times 10^{-5\;\circ }$. From Table 5 it can be seen that, for $\epsilon =1\times 10^{-6}$, the average computation time per sample is $1.012\times 10^{-2}$ s (roughly 99 Hz) and that the average error norm is $7.598\times 10^{-7}$. Both values are adequate given the characteristics of the data. Therefore, we established N=56.

The pseudocode in Algorithm 2 presents the way to solve the inverse kinematics problem for a kinematic chain of *n* DoF. In this work, we use a 3-DoF kinematic chain to model a lower limb on the sagittal plane.

**Algorithm 2** Inverse kinematics**Require:** Target posture coordinates ${\vec{\zeta }}_{g}$, step size factor α, damping factor γ, joint limits $\theta_{i,\min/\max}$, body segments parameters ${\vec{p}}_{i}^{R}$, initial chain configuration ${\vec{\Phi }}^{0}$ and end-effector dual quaternion ${\underline{q}}_{g}^{R}\left({\vec{\Phi }}_{g}\right)$ ϵ←errorthesholdvalue N←maximumnumberofiterations n←numberofjoints ${\vec{\xi }}_{g}^{0}\leftarrow f\left({\vec{\Phi }}^{0}\right)$ k←0 **while**
$|{\vec{\zeta }}_{g}-{\vec{\xi }}_{g}^{k}|>\epsilon$ and k≤N **do**    $J\leftarrow J({\vec{\xi }}_{g}^{k},{\vec{\Phi }}^{k})$    $\Delta {\vec{\xi }}_{g}^{k}\leftarrow \alpha \left({\vec{\zeta }}_{g}-{\vec{\xi }}_{g}^{k}\right)$    $\Delta {\vec{\Phi }}^{k}\leftarrow J^{T}{\left(JJ^{T}+\gamma^{2}I\right)}^{-1}\Delta {\vec{\xi }}_{g}^{k}$    ${\vec{\Phi }}^{k+1}\leftarrow \vec{\Phi }+\Delta \vec{\Phi }$    **for** 
i=1,i=n **do**      ${\vec{\Phi }}_{i,\mathrm{clamp}}^{k+1}\leftarrow \mathrm{clamp}\left({\vec{\Phi }}_{i}^{k+1}\right)$    **end for**    ${\vec{\xi }}_{g}^{k+1}\leftarrow f\left({\vec{\Phi }}_{\mathrm{clamp}}^{k+1}\right)$    k←k+1 **end while** **return**
${\vec{\Phi }}_{\mathrm{clamp}}^{k+1}$

## 3. Results

### 3.1. Inverse Kinematics

**Figure 4:**
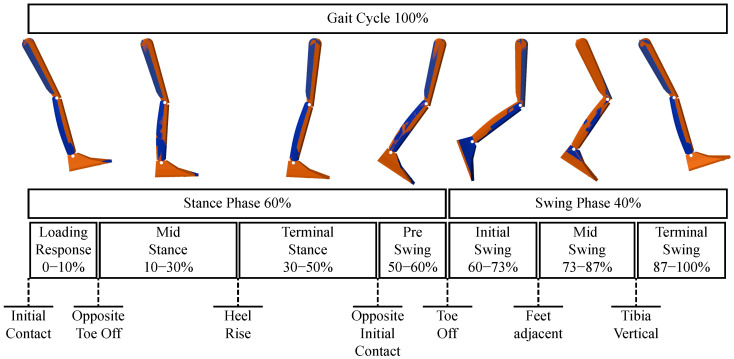
Normal gait cycle reproduced by two superimposed 3D lower limb models using the reference joint angles (blue model) and the estimated joint angles computed through inverse kinematics (orange model).

Figure 4 shows the two 3D models used to illustrate the sequential postures of the body segments during the normal gait cycle in the sagittal plane. The blue model motion is reconstructed from the reference joint angles, while the orange model shows the motion obtained from the estimated joint angles of IK. A visual examination suggests that both models reproduce the gait cycle with minimal differences, resulting in an apparent overlap.

**Figure 5:**
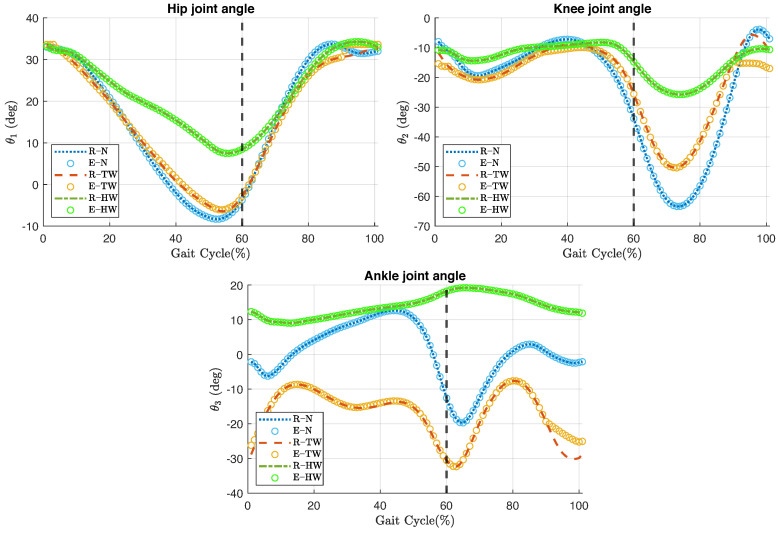
Comparison of the reference (R, dashed lines) and estimated (E, ∘ markers) joint angles for the hip, knee, and ankle joints during normal Gait (N), toe-walking (TW), and heel-walking (HW) cycles. Vertical dashed black line indicates the 60% of the gait cycle.

Figure 5 illustrates the comparison between the trajectories of the reference and the estimated joint angles during the three types of gait. The plots for toe-walking show that, during the last 10% of the gait cycle, the estimated hip joint angle estimation raises to the flexion limit, while the estimated knee and ankle joint angles do not reach the magnitude of the reference angles. As for normal gait and heel-walking, the estimated angles follow the trajectories of the reference angles smoothly, with only slight differences.

The accuracy of the inverse kinematics method is assessed by comparing the estimated joint angles with the reference joint angles using the RMSE as a performance metric. The RMSE of the joint angles is less than $8.407\times 10^{-4\;\circ }$ during normal gait and heel-walking and below $2.6383^{\circ }$ during toe-walking. The highest RMSE corresponds to the knee angles during toe-walking, while the lowest RMSE appears in the hip angles during heel-walking. Table 6 summarizes the RMSE of each joint angle for the different gait types.

A quantitative comparison with a Denavit–Hartenberg (DH) formulation of the DLS algorithm is showed in Table 7. The compared metrics where the average computation time per sample, the average number of iterations per sample, and the average of the three estimated angles’ RMSE. The parameters used for the DH DLS are the same as for the DQ DLS, except for N=100.

### 3.2. Forward Kinematics

In this work, forward kinematics solutions are used to evaluate the effect of the differences between the reference and estimated joint angles on the postures of the anatomical landmarks along the kinematic chain. For this purpose, the trajectories computed by FK_1_ (using reference joint angles) and FK_2_ (using estimated joint angles) are compared using the RMSE of the positions of the anatomical landmarks and the RMSE of the orientations of the body segments.

**Figure 6:**
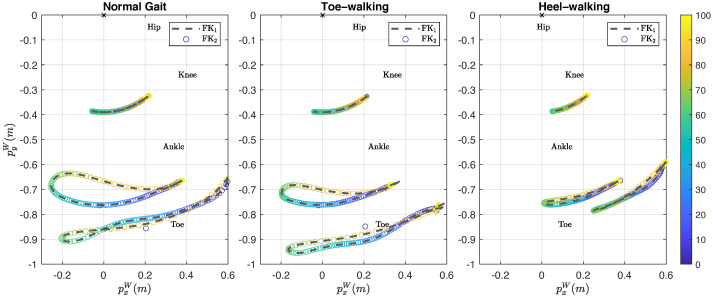
Comparison of the trajectories of the anatomical landmarks using the reference joint angles (${\mathrm{FK}}_{1}$) (dashed line) and the joint angles obtained using inverse kinematics (${\mathrm{FK}}_{2}$) (∘ colorbar). Where, the vertical right colorbar is the percentage of the gait cycle.

Figure 6 presents a comparison between the Cartesian coordinates on the sagittal plane, computed by FK_1_ and FK_2_, for each anatomical landmark across the three types of gait. As expected from the IK results, there are no significant differences in the trajectories of the anatomical landmarks during normal gait and heel-walk. However, the trajectories of the ankle and toe landmarks computed by FK_2_ show noticeable deviations from those of FK_1_ during the final part of the cycle.

The RMSE for the positions are below $2.592\times 10^{-6}$ m during normal gait and heel-walk, and below $11.1\times 10^{-3}$ m during toe-walk. The highest RMSE corresponds to the toe position during toe-walking, while the lowest RMSE appears in the toe position during normal gait. Table 8 summarizes the RMSE values for the position of the anatomical landmarks for each gait type cycle.

**Figure 7:**
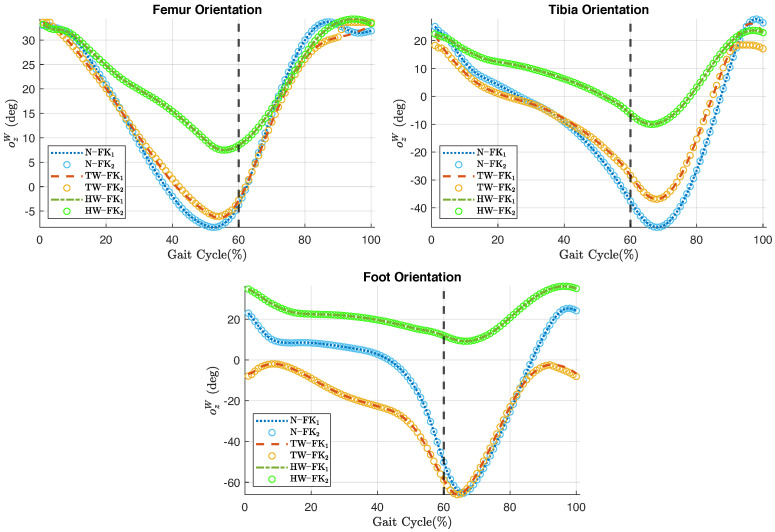
Comparison of the orientation of the body segments with respect to the global coordinate system during normal gait (N), toe-walking (TW), and heel-walking (HW) cycles; calculated using FK_1_ (dashed line) and FK_2_ (∘ line). Vertical dashed black line marks the 60% of the gait cycle.

Figure 7 shows the comparison of the body segments’ orientations during the three gait types calculated by FK_1_ and FK_2_. Femur and tibia orientations during toe-walk show visible differences during the last 10% of the cycle. The plots also show that the differences in foot orientation during the last part of the toe-walking cycle are less noticeable, as indicated by the RMSE value in Table 9.

The orientation RMSE during normal gait and heel-walk are below $4.592\times 10^{-4\;\circ }$, while the RMSE for toe-walk reaches $2.0105^{\circ }$ (Table 9). The highest RMSE corresponds to the tibia orientation during toe-walking, while the lowest RMSE appears in the foot orientation during heel-walking. It is important to note that since the body segments of the three-DoF model rotate within a 2D plane, only rotations about the $o_{z}^{W}$ axis are analyzed.

## 4. Discussion

In previous works we addressed the solution of the forward kinematics problem to determine only the position of anatomical landmarks [26] and a geometric approach to estimate the joint coordinates [17]. This was useful for a first approximation but results in an incomplete description. In order to provide a complete solution, that is, a solution that not only describes the anatomical landmark positions at the knee, ankle, and hip but also that quantifies the angular variations of the joints and body segments in the sagittal plane, in the present study two main objectives were set. First, it aims to develop a framework for kinematic gait analysis using a dual-quaternions composition to solve the forward kinematics problem and the DLS Jacobian method for the inverse kinematics problem. Second, it seeks to assess whether the proposed method is applicable to different types of gait whose characteristics resemble those seen in pathological gait patterns.

Regarding the development of the proposed framework, the algebraic formulation presented in Section 2 supports the reliability of the forward kinematics solution. Other representation formalisms, such as unit quaternions, Euler angles, and angle–axis representations in combination with 3D vectors, describe rotation and translation independently, which may result in a loss of coupling between rotation and translation [41]. In contrast, dual-quaternion operations have been widely demonstrated to provide a unified and formally valid representation of rigid body transformations in three-dimensional Euclidean space, effectively integrating rotation and translation [23]. Dual quaternions also avoid discontinuities and singularities that arise from the Euler angle representation, such as the phenomenon of gimbal lock [42], and can be used to generate fast reliable IK solutions in real-time for highly articulated models [43].

Furthermore, dual quaternions offer some advantages in representing human motion in gait analysis compared to homogeneous transformation matrices. Dual quaternions can be easily concatenated, interpolated smoothly, and provide rigid transform comparisons effortlessly [43], but more important, they are a compact representation, using only eight parameters to represent both rotation and translation, while homogeneous matrices require twelve parameters [42], which also reduces the number of algebraic operations for kinematic modeling applications [41].

On the other hand, Figure 5, Figure 6 and Figure 7 serve to highlight the differences in joint angles, anatomical landmark positions, and segment orientations over the three types of gait analyzed. In toe-walking, hip extension and knee flexion are reduced, and ankle dorsiflexion is absent. While, heel-walking is characterized by a lack of hip extension, reduced knee flexion, and decreased ankle plantarflexion. These gait abnormalities are features of pathological gaits seen in disease conditions such as cerebral palsy [44], Parkinson’s disease [45], and post-stroke hemiplegia [46]. Gait deviations exhibited in impairments such as the aforementioned tend to be a continuum of deviations rather than distinct categories. Therefore, an accurate description of individual gait deviations, rather than a group classification, may provide better guidance for the development and customization of therapeutic treatments.

The RMSE data summarized in Table 6, Table 8, and Table 9 show that, in general, both the estimated angles and the computed end-effector postures are close to the reference joint angles and to the target postures, respectively, for all gait types analyzed. Consequently, we consider that the proposed approach holds potential for gait analysis in clinical settings and that it can be applied to identify and quantify motor deficits, as well as to provide an objective assessment of gait treatments [47].

Quantitative comparison of the proposed DLS algorithm with a Denavit–Hartenberg formulation shown in Table 7 exhibit an improvement in computation time per sample, and a reduction in the number of iterations and on the average of the RMSE of the estimated angles in all gait types except for the toe-walking pattern. Therefore, further optimization of the algorithm and its parameters must be realized in order to solve the visible problems that limit the current performance of the proposed method.

### Limitations

The presented IK method performs the estimations of the joint angles based only on the foot segment postures. Although in some cases this does not make it difficult to obtain an anatomically correct IK solution; in some other cases this results in the need for conditions to force a solution with a forward knee configuration and ensure fast convergence time at the beginning of the estimation process. The first condition is the need to impose an initial posture different from the anatomical posture. The initial postures used in this work are based on the rotation of the joints in the first step of the gait pattern analyzed. Currently, there is no general posture that is applicable for all different types of gait, but that would be desirable. In fact, in the case of heel-walking, to ensure fast convergence time at the beginning of the estimation process, it was necessary to set the initial estimated posture very close to the initial target posture.

The second condition is the need to impose joint limits close to the limits of the specific range of motion of the gait type being analyzed. The toe-walking gait cycle is an example of this scenario. A close look at Figure 5 reveals a deviation of the estimated angles during the last 10% of the toe-walking gait cycle. The effects of this angular deviation are reflected in the positions of the anatomical landmarks and in the orientations of the body segments, as shown in Figure 6 and Figure 7, respectively. Although the imposition of joint limits helped to considerably reduce this deviation, it was not possible to eliminate it completely.

## 5. Conclusions

In this paper we presented a framework for solving forward and inverse kinematics problems, applied to two-dimensional gait analysis of a three-DoF model of the human lower limb. However, due to the generality of the formulation, the method can be extended to the analysis of kinematic chains with more degrees of freedom and multiple end-effectors, as would be the case of a model of the human body representing the motion of all its limbs in three dimensions. Furthermore, we consider that the proposed algorithms could be used in diverse scenarios such as performance evaluation in sports or in the clinical diagnosis of motor deficits. In particular, the results demonstrate that diseases that share characteristics with toe-walking and heel-walking, such as cerebral palsy, Parkinson’s disease, or post-stroke hemiplegia could be analyzed using the proposed method.

Despite the encouraging results, a rigorous comparison with other numerical methods and IK solvers is still needed. In addition, a review of novel methods, such as those based on machine learning, could provide useful tools to generalize the results and reduce the need for specific parameterizations for each type of motion. Finally, it is necessary to consider the inclusion of more diverse data and therefore the development of the necessary methods for the acquisition and processing of data from different types of sensors for the recording of gait kinematic data.

## Figures and Tables

**Table 1:** Parameters of the body segments.

Parameter	Length
Body height	1.65 m
Femur length	0.37 m
Tibia length	0.38 m
Lateral malleolus height	0.08 m
LM-to-DPH	0.25 m

**Table 2:** Angular limits of the joints.

Motion	Limit	Normal Gait	Toe-Walking	Heel-Walking
Hip flexion	$\theta_{1,\max}$	$180^{\circ }$	$33.6^{\circ }$	$34.3^{\circ }$
Hip extension	$\theta_{1,\min}$	$-180^{\circ }$	$-6.4^{\circ }$	$7.5^{\circ }$
Knee extension	$\theta_{2,\max}$	$180^{\circ }$	$-5.6^{\circ }$	$-8.2^{\circ }$
Knee flexion	$\theta_{2,\min}$	$-180^{\circ }$	$-50.4^{\circ }$	$-25.8^{\circ }$
Ankle dorsiflx.	$\theta_{2,\max}$	$180^{\circ }$	$-7.6^{\circ }$	$19.2^{\circ }$
Ankle plantarflx.	$\theta_{2,\min}$	$-180^{\circ }$	$-32.3^{\circ }$	$9^{\circ }$

**Table 3:** Initial chain configuration values.

Joint	Normal Gait	Toe-Walking	Heel-Walking
Hip	$1^{\circ }$	$1^{\circ }$	$32^{\circ }$
Knee	$-5^{\circ }$	$-5^{\circ }$	$-5^{\circ }$
Ankle	$-1^{\circ }$	$1^{\circ }$	$12^{\circ }$

**Table 4:** IK algorithm parameter values.

Parameter	Normal Gait	Toe-Walking	Heel-Walking
α	0.6992	0.7334	0.7301
γ	0.0579	0.0608	0.0579

**Table 5:** Relationship between ϵ, the number of iterations, and the error values.

ϵ	Avrg. Time per Sample (s)	Min. of Iterations	Max. of Iterations	Avrg. Error Norm
$1\times 10^{0}$	$1.482\times 10^{-4}$	1	2	$4.355\times 10^{-1}$
$1\times 10^{-1}$	$2.956\times 10^{-4}$	1	4	$5.824\times 10^{-2}$
$1\times 10^{-2}$	$1.624\times 10^{-3}$	1	18	$5.164\times 10^{-3}$
$1\times 10^{-3}$	$2.714\times 10^{-3}$	3	34	$6.985\times 10^{-4}$
$1\times 10^{-4}$	$4.905\times 10^{-3}$	5	79	$7.425\times 10^{-5}$
$1\times 10^{-5}$	$7.672\times 10^{-3}$	5	35	$7.465\times 10^{-6}$
$1\times 10^{-6}$	$1.012\times 10^{-2}$	10	56	$7.598\times 10^{-7}$
$1\times 10^{-7}$	$1.307\times 10^{-2}$	12	83	$7.336\times 10^{-8}$
$1\times 10^{-8}$	$1.623\times 10^{-2}$	14	125	$7.526\times 10^{-9}$
$1\times 10^{-9}$	$1.942\times 10^{-2}$	16	167	$7.591\times 10^{-10}$

**Table 6:** RMSE of the comparison between the references and estimated joint angles.

Joint	Normal Gait	Toe-Walking	Heel-Walking
Hip	$3.825\times 10^{-4\;\circ }$	$0.6593^{\circ }$	$2.558\times 10^{-4\;\circ }$
Knee	$8.407\times 10^{-4\;\circ }$	$2.6383^{\circ }$	$6.234\times 10^{-4\;\circ }$
Ankle	$4.584\times 10^{-4\;\circ }$	$1.6130^{\circ }$	$3.661\times 10^{-4\;\circ }$

**Table 7:** Comparison between Denavit–Hartenberg (DH) and dual quaternion (DQ) formulation for joint angles estimation using DLS algorithm.

Metric	Normal Gait DQ	Normal Gait DH	Toe-Walking DQ	Toe-Walking DH	Heel-Walking DQ	Heel-Walking DH
Avg. time per sample	0.011	0.063	0.019	0.062	0.0102	0.054
Avg. n. of iterations	19	96	35	95	20	90
Avr. angle error	$5.6054\times 10^{-4\;\circ }$	$8.3113\times 10^{-4\;\circ }$	2.167	$3.704\times 10^{-4\;\circ }$	$4.142\times 10^{-4\;\circ }$	$5.483\times 10^{-4\;\circ }$

**Table 8:** RMSE of the position comparison of the anatomical landmarks.

Anatomical Landmark	Normal Gait	Toe-Walking	Heel-Walking
Knee	$2.592\times 10^{-6}$ m	$4.4\times 10^{-3}$ m	$1.743\times 10^{-6}$ m
Ankle	$0.724\times 10^{-6}$ m	$9.8\times 10^{-3}$ m	$0.810\times 10^{-6}$ m
Toe	$0.708\times 10^{-6}$ m	$11.1\times 10^{-3}$ m	$0.807\times 10^{-6}$ m

**Table 9:** RMSE of the comparison of the orientation of the body segments.

Body Segment	Normal Gait	Toe-Walking	Heel-Walking
Femur	$3.808\times 10^{-4\;\circ }$	$0.6531^{\circ }$	$2.560\times 10^{-4\;\circ }$
Tibia	$4.592\times 10^{-4\;\circ }$	$2.0105^{\circ }$	$3.685\times 10^{-4\;\circ }$
Foot	$0.198\times 10^{-4\;\circ }$	$0.4456^{\circ }$	$0.045\times 10^{-4\;\circ }$

## Data Availability

The original contributions presented in this study are included in this article; further inquiries can be directed to the corresponding author.
